# Reduced performance after return to competition in ACL injuries: an analysis on return to competition in the ‘ACL registry in German Football’

**DOI:** 10.1007/s00167-022-07062-8

**Published:** 2022-07-10

**Authors:** Dominik Szymski, Leonard Achenbach, Johannes Weber, Lorenz Huber, Clemens Memmel, Maximilian Kerschbaum, Volker Alt, Werner Krutsch

**Affiliations:** 1 Department of Trauma Surgery, University Medical Centre Regensburg, Franz-Josef-Strauss Allee 11, Regensburg, Germany; 2 FIFA Medical Centre of Excellence, University Medical Centre Regensburg, 93053 Regensburg, Germany; 3 Department of Orthopedics, König-Ludwig-Haus, Julius-Maximilians-University Wuerzburg, Würzburg, Germany; 4 Department of Pediatric Surgery and Pediatric Orthopedics, Clinic St. Hedwig Regensburg, Regensburg, Germany; 5 SportDocs Franken, Nuremberg, Germany

**Keywords:** Return to play, Athletic injury, Sports medicine, ACL, Knee injury, Team sport, Rehabilitation

## Abstract

**Purpose:**

ACL injuries are one of the most severe injuries in football, but medical consequences and performance outcomes after return to competition are only rarely investigated. Aim of this study was to analyse the time of return to competition (RTC) in German professional, semi-professional and amateur football. Also, this investigation highlights the rate of career ending and performance outcome after RTC in different playing levels by the measurement of playing level, performed matches and played minutes.

**Methods:**

Database of this investigation is the ‘ACL registry in German Football’ with prospectively collected injury data. Between 2014 and 2018, four seasons in professional (1st–3rd league), semi-professional (4th–6th league) and amateur leagues (7th league) were analysed regarding the return to competition period and performance parameters. Data were collected for three subsequent seasons after injury and compared with the pre-injury and injury season. Data collection was performed using standardized methods.

**Results:**

A total of 607 ACL injuries were registered during the 4-year period with a mean RTC time of 337.1 day (SD: 183). After primary ACL ruptures, the fastest RTC was found in professional football (247.3 days), while in semi-professional (333.5 d; *p* < 0.0001) and amateur football (376.2 d; *p* < 0.0001) a prolonged absence was detected. Re-ruptures occurred in 17.8% (*n* = 108) and showed similar trend with fastest RTC in professionals (289.9 days; *p* = 0.002). Within the first three seasons after injury, 92 players (36.7%) in semi-professional and 24 (20%) in professionals had to end their career. Keeping the level of play was only possible for 48 (47.5%) of professionals, while only 47 (29.6%) of semi-professionals and 43 (28.1%) of amateurs were able to. Only in professional football, no significant difference could be seen in the played minutes and games after 2 years compared to the pre-injury season.

**Conclusion:**

Lower playing levels and re-ruptures are the main factors for a prolonged return to competition after ACL rupture in German football. Significant reduction in playing level and a high rate of career endings were found for all levels of play. However, only professional players were able to regain their playing minutes and games 2 years after injury, while lower classed athletes did not reach the same amount within 3 years.

**Level of evidence:**

Level III.

## Introduction

Anterior cruciate ligament (ACL) injuries belong to one of the most severe lesions in sports medicine. These injuries mainly concern young athletic men and lead to a high number of sick-leave days in sports but also in the professions of affected athletes and therefore to high personal and socio-economic costs [5]. Parallelly, ACL injuries are one of the most common causes of permanent disability in younger patients and associated with an increased risk of osteoarthrosis [4, 9]. In particular, in football the incidence of ACL ruptures is increased compared to other team sports and therefore considered as “high-risk” sport [22]. Amateur players are thereby in charge of an increased risk with a significantly higher probability of occurrence [3, 21, 26]. Majority of all football players (est. over 450 million worldwide) are accounted as recreational athletes and suffer from this increased risk of sustaining an ACL rupture [26]. With a season prevalence of 12.1 (range 6.8–15.9) ACL injuries for a recreational league and overall 2235 football leagues in Germany (https://www.dfb.de/), number of around 27,000 (range 15,198–35,536) cruciate ligament ruptures due to playing and training activities in football can be expected in Germany every year [26, 27]. Particularly for amateur players, a loss of time after an anterior cruciate ligament rupture with subsequent rehabilitation and a high number of sick-leave days mean an enormous burden [5]. While the injury mechanism in football is already well described as a mainly non-contact or indirect contact injury after tackling or pressing situation [2, 3] and risk factors are defined [21, 26], detailed information about the rehabilitation and long-term effects on performance are missing for this level of performance. For professional players, sufficient data on rehabilitation and recovery after ACL rupture are available. However, majority of this research was performed in elite teams playing international tournaments and showed already a low rate of return to same performance level [6, 23, 29]. For the first league in Germany (Bundesliga), a mean time of return to competition of 226.7 days after primary rupture and 245.6 days after re-rupture were described by a previous investigation [13]. For amateur and semi-professional players, who build the majority of injured players and suffer from an increased risk, no data on rehabilitation times and return to competition are available and build a lack in scientific literature.

The aim of this study was the analysis of return to competition time between different playing levels in order to highlight the specific aspects in different leagues. The lack of return to play data in semi-professional and in particular in amateur football should be addressed with this investigation. Mid-term and long-term performance outcomes in different levels of play were as well analysed for a period of five years, beginning with the pre-injury season till three years after injury.

## Materials and methods

### Study population and design

The study design of the National ACL registry in football was approved by the Ethics Committee of the University of Regensburg (ID: 10-37_5-101). This retrospective register study with prospectively collected data investigates ACL injuries in all professional football clubs in Germany and all non-professional clubs of the Bavarian Football Association in men’s football with regard to return to competition time and match performance for three seasons after injury. Injury data were registered in a standardized manner in the prospective nationwide ACL registry in German football since the 2014–15 season [27]. All male football players with a new ACL injury who had actively played in the German men's professional leagues (1st–3rd men’s professional league) or in the Bavarian amateur leagues (4th–7th men’s amateur league) were included into the study population. For this investigation, all ACL injuries in 4 consecutive seasons from 2014–15 to 2017–18 were analysed in the mentioned leagues. Performance of the players was registered for the 3 subsequent seasons after injury and compared to the season before injury and injury season. All levels from the 1st to the 6th league and 8 of the 12 leagues of the 7th playing level of men’s football were included for further analysis. Four leagues of the 7th playing level were excluded from analysis because a lack of injury data. The study population was divided into men’s professional (1st–3rd league), semi-professional (4th, and all 5th and 6th leagues) and amateur football (7th leagues). Professional football players were defined as full-time athletes with a contract, a salary and full insurance and semi-professional football players as paid players with a contract and insurance but no sufficient income to live on.

### Injury documentation and data collection

Annually football officials, clubs and team physician were informed and invited to participate in the ACL registry in German Football and report new ACL ruptures to our study office. Parallelly, a double-check of the registration data by means of national media data was obtained by the study team by a standardized data collection method to avoid typical data loss and to decrease the drop-out rate [27]. Krutsch et al ([12, 13]) validated the double-check system for media analysis for ACL injuries in football, where 100 % of injuries could be confirmed [12, 13]. On the basis of these ACL registration data after each season, official match statistics were analysed in regard to return to competition characteristics of the injured player. Thereby the time of return to competition was detected by analysis of official match records and compared with the registered injury date. Concurrent the match performance was recorded by registration of playing level and total amount of officially played matches as well as match minutes. These data were recorded retrospectively for one season before and the season of the ACL rupture and prospectively for 3 seasons after injury. Thus, a record of match performance over the course of 5 seasons was generated.

The injury reports for this study were adapted according to the commonly used injury report protocol in football established by Fuller et al (2006) and according to previous epidemiological injury studies of this study group [7, 14, 17, 25].

### Statistical analysis and data assessment

Categorial data are expressed as frequency counts (percentages) and continuous data as mean ± standard deviation (SD). Proportions between groups were compared with the Fisher’s exact test and continuous variables with the *t* test. This register was about comparing different leagues in terms of return to play and other parameters, without prior knowledge about possible effects and therefore no formal case number calculation or power analysis was performed. For this purpose, all players were extracted from the register in order to achieve the maximum possible power. The study office used the RedCap-System for data management and IBM SPSS Statistics, version 26.0 for data analysis.

## Results

In the study period between 2014 and 2018, a total of 607 verified total ACL injuries occurred in all investigated men football leagues. Twelve injuries revealed to be partial lesions of the ACL and were excluded. Four hundred and nighty-nine (82.2%) of the injuries were primary, and 108 (17.8%) re-ruptures. Majority of injuries were registered in semi-professional football players (*n* = 251; 41.3%), while in amateur 236 (38.9%) and in professional football 120 (19.8%) ruptures were detected (Table 1).

**Table 1:** Anthropometric data of the study population

	Professional *n* = 120	Semi-professional *n* = 251	Amateur *n* = 236	Total *n* = 607
	*n* ± SD (min; max)	*n* ± SD (min; max)	*n* ± SD (min; max)	*n* ± SD (min; max)
Age in years	24.7 ± 4.3 (18; 32)	24.1 ± 3.9 (18; 40)	25.5 ± 4.2 (18; 45)	24.7 ± 4.1 (18; 45)
Weight in kg	79.4 ± 7.4 (52; 98)	77.1 ± 6.7 (64; 101)	79.9 ± 15.3 (63; 121)	78.7 ± 10.8 (52; 121)
Height in cm	182.7 ± 6.7 (157; 200)	181.2 ± 5.8 (170; 198)	180.1 ± 11.6 (166; 200)	181.4 ± 8.4 (155; 200)
BMI in kg/m^2^	23.6 ± 1.3 (19.0; 26.9)	23.4 ± 1.7 (19.1; 28.7)	27.0 ± 3.1 (19.9; 33.8)	24.8 ± 2.1 (19.0; 33.8)
Experience in years	18.9 ± 3.9 (10; 28)	18.2 ± 3.9 (7; 26)	19.8 ± 4.7 (3; 35)	19.0 ± 4.3 (3; 35)

**Fig. 1:**
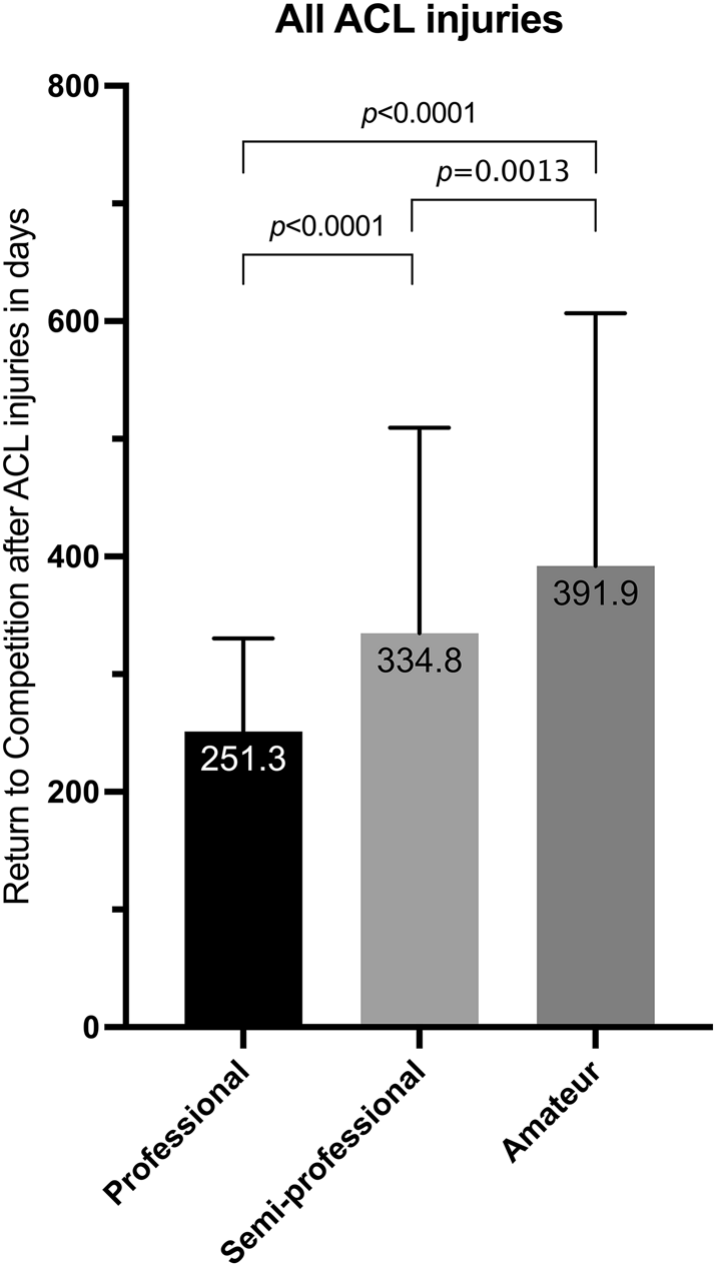
Return to competition (RTC) time after ACL injuries (cumulated for primary ruptures and re-ruptures) in different levels of play in Germany

Overall, in football a mean return to competition of 337.1 days (SD: 183.0) was detected during the study period. Fastest RTC was found in the 3rd league with a mean loss of 239.4 days, while in amateur football (7th league) the longest time period between injury and return to competition (mean: 391.9 days) was measured. Professional football showed compared to semi-professional (*p* < 0.0001) and amateur football (*p* < 0.0001) significant faster return to competition in the cumulated time periods (Fig. 1). A difference by trend was found under the sub-group of professional players, where the 3rd league (239.4 days; SD: 70.4) had a faster return to competition than in the first league (n.s.) or 2nd league (n.s.) (Table 2).

**Table 2:** Return to competition time in different levels of football after ACL injury

	Number of ACL injuries	Mean return to competition (in days)	SD	Min; max
1st league	37	252.3	± 71.2	134; 470
2nd league	33	268.8	± 97.2	171; 701
3rd league	50	239.4	± 70.4	105; 448
Professional football	**120**	**251.3**	** ± 79.0**	**105; 701**
4th league	46	324.1	± 224.5	139; 1504
5th league	75	299.9	± 111.1	123; 634
6th league	130	358.8	± 181.6	98; 1245
Semi-professional football	**251**	**334.8**	** ± 174.7**	**98; 1504**
Amateur football	**236**	**391.9**	** ± 214.8**	**116; 1348**
Total	**607**	**337.1**	** ± 183.0**	**98; 1504**

**Fig. 2:**
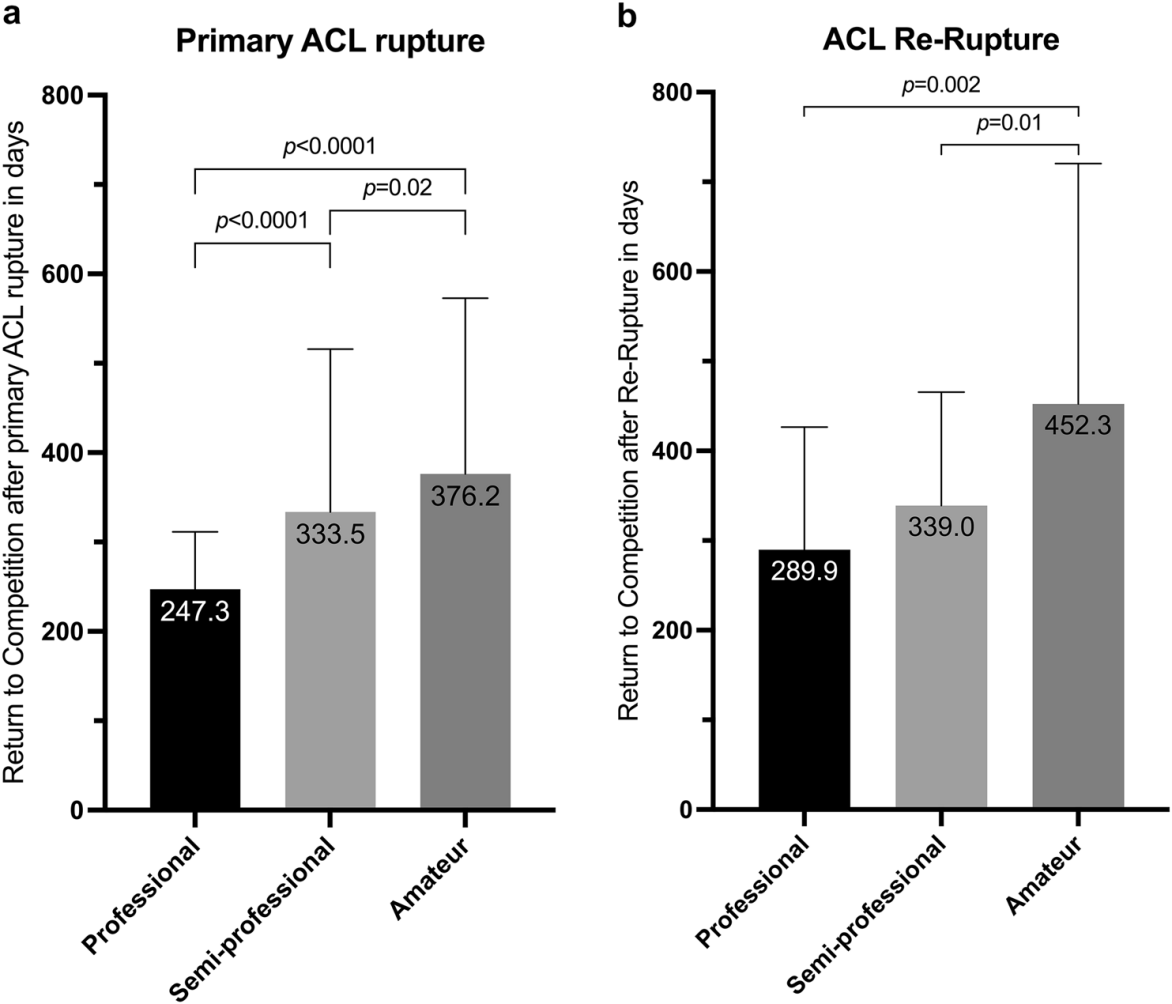
Return to competition time in different levels of play in Germany after primary (**A**) ACL rupture and ACL re-rupture (**B**)

After primary ACL injury, the fastest return to competition was found in professional football with a mean time loss of 247.3 days compared to 333.5 days in semi-professional (*p* < 0.0001) and 376.2 days in amateur football (*p* < 0.0001). However, for ACL re-ruptures no significant difference was registered between professional and semi-professional football, but an increased time until resumption of match activity in amateur football (452.3 days; professional: *p* = 0.002; semi-professional: *p* = 0.01 (Fig. 2).

**Fig. 3:**
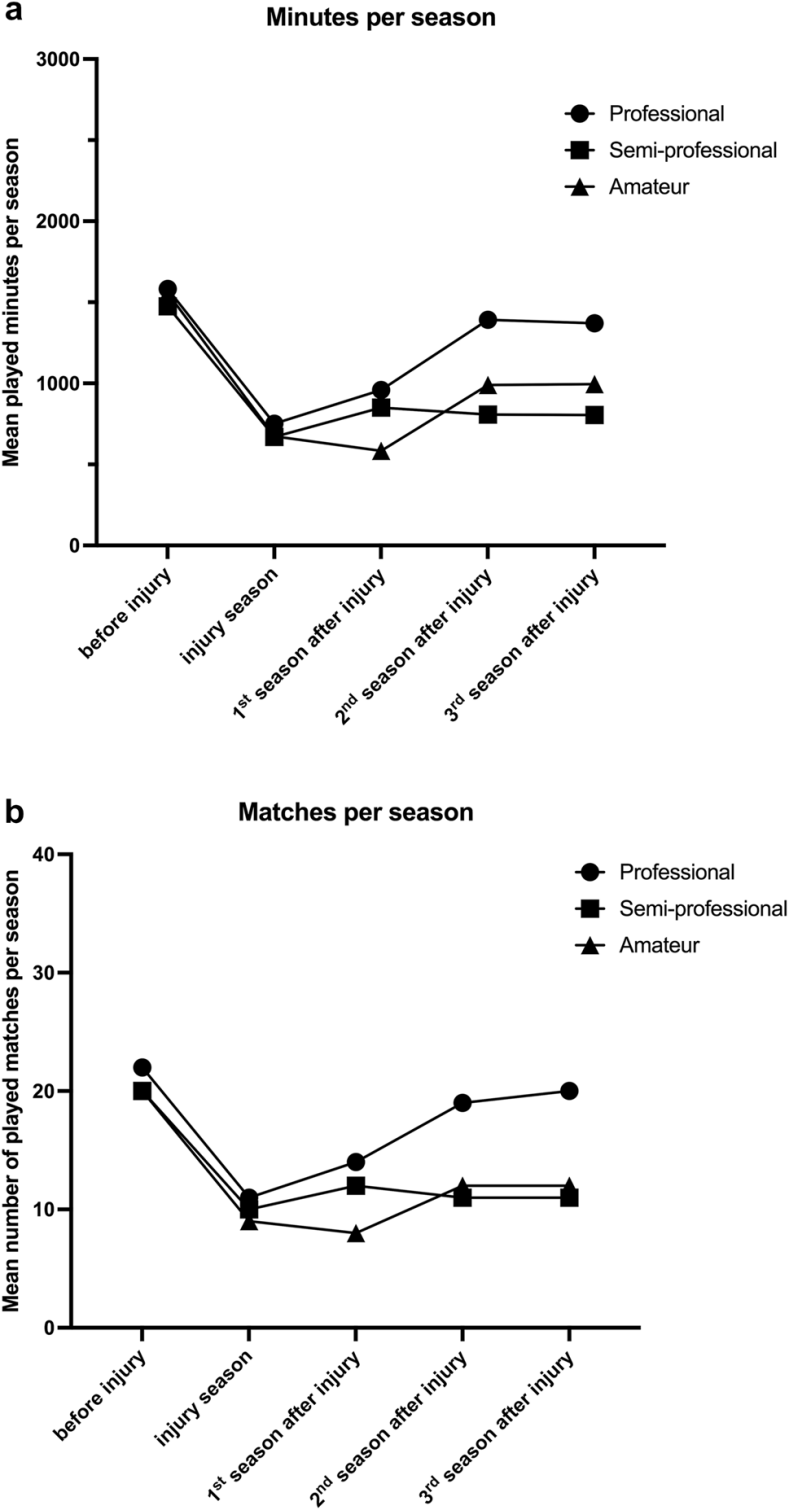
Development of played minutes (**A**) and number of played matches (**B**) in the season before ACL injury, the season of injury and three subsequent seasons in German football

Two seasons after the anterior cruciate ligament injury, the average playing time in minutes (1392 ± 992 min) in professional football was no longer significantly different from the pre-injury season (1582 ± 887 min; n.s.). However, in semi-professional and amateur football in the third season after ACL injury still a significant (*p *< 0.0001) difference to the pre-injury season was documented for the remaining active athletes. Equal pattern was detected for played matches within this time period (Fig. 3).

**Fig. 4:**
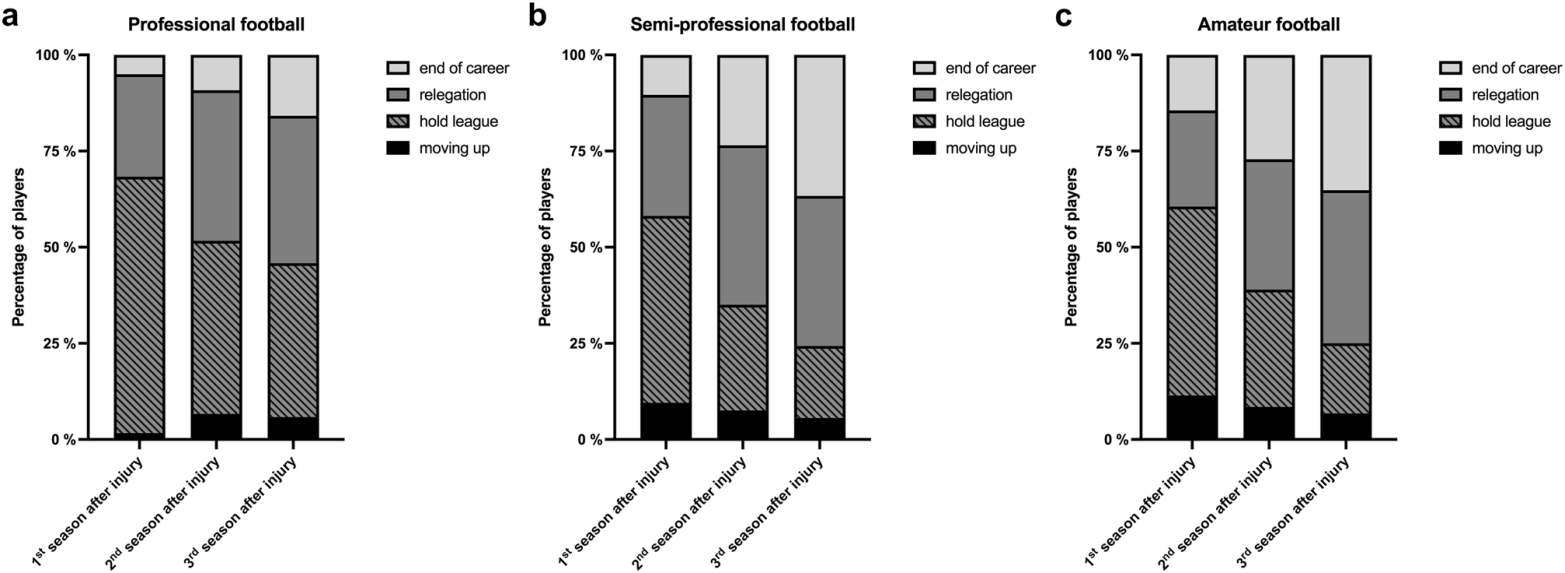
Development career endings and levels of play compared to the pre-injury season within a time period of three seasons after ACL injury in professional (**A**), semi-professional (**B**) and amateur (**C**) football

The ACL re-rupture rate was between 15.0 % (professional) and 20.8 % (amateur). Within the first three seasons, 24 professional players (20.0 %) and 83 amateur football players (35.2 %) had to quit their career. In all levels of play, the proportion of players having to descend from their level of play in the pre-injury season was reported around 39 %. In professional football 48 (50.0 %), in semi-professional 47 (22.4 %) and in amateur football 43 (23.0 %) of players could hold their level of play after three years (Table 3). With every season following the ACL injury, the proportions of career endings and relegations are increasing in all performance groups (Figure 4). Subsequent injuries with a time loss appeared in 35.0 % of the professional athletes and led to a mean loss of 84.3 days, while in amateur football 23.7 % subsequent injuries were reported in the first season after ACL rupture (Table 3).

**Table 3:** Performance effects within the first three seasons after ACL injuries in football players

	Professional football (*n* = 120)	Semi-professional football (*n* = 251)	Amateur football (*n* = 236)	Total (*n* = 607)
	*n* (%)	*n* (%)	*n* (%)	*n* (%)
ACL Re-rupture	18 (15.0)	41 (16.3)	49 (20.8)	**108 (17.8)**
End of career within 3 seasons after injury	24 (20.0)	92 (36.7)	83 (35.2)	**199 (32.8)**
Relegation in league within 3 seasons after injury	46 (39.2)	98 (39.0)	94 (39.8)	**144 (39.2)**
Any subsequent time-loss injury within the first season after ACL injury	42 (35.0)	46 (18.3)	56 (23.7)	**144 (23.7)**
Mean ± SD (min; max)				
RTC after other subsequent injury	84.3 ± 140.7 (0; 663)	46.0 ± 107.3 (0; 695)	230.7 ± 193.4 (7; 723)	**59.1 ± 128.4 (0; 723)**

## Discussion

Main finding of this register study about ACL injuries in football was the determination of the return to competition time after an anterior cruciate ligament injury in German football at all levels of play. Thereby a clear significant trend of prolongation of rehabilitation time was detected with decreasing level of play for primary ACL ruptures as well as ACL re-ruptures. Another major insight of this study was the documentation of match performance within the first three seasons after ACL rupture compared to the pre-injury season. This showed a concerning high proportion of players in all rules who were unable to maintain the level of play or were forced to end their careers.

Previous investigations with relation to the time period between ACL injury and return to competition in football focused mainly on the professional levels in Europe. Within a timeframe of 20 years, Forsythe et al. analysed 51 ACL injuries in European elite football and reported a mean RTP of 215.5 days and 25 missed matches [6]. In the first league in Germany as well a loss of 226.7 days after primary and 245.6 days after re-rupture were already documented [13]. Other studies concerning the elite teams of European football showed a return-to-competition period of 225.0 and 209.0 days, respectively [23, 29]. However, these data only cover the international acting teams of European top leagues. In our investigation, a longer return to competition was detected in the first league (Bundesliga) with a mean RTC of 252.3 days. An interesting trend is found in the professional classes (1st–3rd league), where shorter downtimes were documented with deceasing level of play. We also found an analogical trend with increasing incidence rates for ACL ruptures with decreasing level of division in the professional classes, with highest incidence rates in the 3rd league [26]. Lower professional classes are often the first step for younger player, where they have to prove themselves and are faced with increased training and match intensity [14]. While already for athletes in leagues below the first division a lack of data was present, in amateur classes even less data are available for the return-to-competition measurement in football players after ACL rupture. Brophy et al. ([1]) reported in 55 male football players a mean RTC of 10.2 months, but did not mention a detailed classification of playing level [1]. Equal results are published in a functional magnetic resonance imaging study after ACL reconstruction in football players, where the mean RTP was 10.0 months [28]. Apart from these studies with 55 and 26 participants each, there are no data on return to competition after ACL rupture in semi-professional and amateur football. We summarized almost 500 semi-professional and amateur players in our investigation and can report a clear trend of longer return to competition with decreasing level of the appropriate league. Thereby a significant difference was documented for the group of semi-professionals (4th–6th league) to amateur (7th league and below) for primary ruptures, as well as re-ruptures. An important result was the mean time of 13 months of RTC in amateur players.

The return to competition in football after ACL rupture is a controversial discussion between players, trainers and physicians. Often the athlete does not agree with physicians’ opinion on the return-to-competition date. Loose et al. (2018) showed in professional and semi-professional played football in Germany already a proportion of 64.4% of decision making against physicians recommendation in regards of return to competition [16]. However, there is clear evidence that an increased risk of re-rupture is reported for a too fast return to sport.

Grindem et al. detected in [8] a reduction in re-rupture risk by 51% for each month delay. From the 9th month after surgery onwards, no significant reduction in the risk due to delay of the return to sport was observed [8]. As a sufficient tool for the decision about return to sport and competition test, batteries measuring muscle strength and functional parameters could be used. Kyritsis et al. ([15]) demonstrated a significant association of re-ruptures with a failure in at least one of six tests of the battery and decreased hamstring-quadriceps ratio [15].

Official records offer data for all levels of performance and make it able to track the athletes performance [12]. Previous works in professionals mentioned already the shocking proportion of 11.8–20% players having to end their career within three years after injury [6, 13, 23, 29]. In all three investigated professional divisions in our study, the same pattern was detected. However, in semi-professional and amateur classes a more alarming result was found. Here even 36.7 and 35.2%, respectively, have to end their career within the first three years after ACL injury with constantly growing rates from the first season after RTC. The only data on amateur player highlighted after a mean follow-up of 7.2 years in 55 athletes a rate of 62% career endings [1]. In professional football players in 62.9% of retired athletes, an injury was the cause of the end of career with injuries mainly affecting the knee and ankle [11]. For amateur players, we expect a similar or even higher proportion, which is conducted to our results on only ACL injuries. Mai et al. ([18]) demonstrated also in the USA for the high-risk sports American football, ice hockey, basketball and baseball also a proportion of only 67% of athletes being active three years after ACL injury [18].

Compared to the pre-injury level, we found for all levels of play a proportion of around 40% of players, who played at in least one division lower. For professional athletes literature describes similar results [6, 13, 29], whereas in amateur players no data are available. While professionals can hold their level of play in around 50% of the cases in the first three years after ACL injury, semi-professionals and amateur players suffer from low rates of players in the same league (semi-professional 22.4%; amateur 23.0%). The Panther Symposium on ACL Injuries described in a Consensus Statement as one main goal of the return to sport after ACL rupture the restorage of the previous performance level [20]. The high difference between professional and semi-professional or amateur players highlights the differences in rehabilitation capacity and medical support of the athletes. In particular, longitudinal care and peer-reviewed test batteries to determine sports ability are decisive factors. A structured rehabilitation plan helps the patients, the therapists, team coaches and all involved trainers. Especially at lower levels of performance, physical therapists or team trainers often have to take on multi-cellular tasks and also have a tremendous impact on the player's rehabilitation. The final decision regarding full return to play should be made in a multidisciplinary manner with orthopaedic surgeons, therapists and coaches involved and should be based on objective criteria (e.g. examinations, RTS test batteries) [19, 20].

In the analysis of played minutes and matches, the alarming trend of performance reduction in the performance groups below professional players was confirmed. In professionals, no significant difference was found in two years after ACL injury as well in our results and in the study of Forsythe et al. [6].

However, semi-professionals and amateur athletes do not reach in the first three years following injury the pre-injury level of played minutes and matches. A clear deficit in divisions below the professional classes was registered with regard to the rehabilitation time, level of performance, played minutes and matches. Besides a reduced physical condition, a parallel burden on these athletes caused by a primary employment and a worse and less frequent medical tracking are potential influences [10, 24]. A more frequent tracking of injured players and supervision during the rehabilitation by the treating physician could help to reduce the high rates of performance reduction in semi-professionals and amateur footballers.

Besides multiple benefits, this analysis of data from the national ACL registry has also some limitations. The data basis was assessed by the ACL registry and a double-check registration of injuries through personal notice by the player or team and media based check by the study office [27]. Potentially, injuries cannot be recorded in this process. However, the aim of the study was not to show the probability of occurrence but to follow-up on ACL injuries, so that this limitation is negligible. Another limitation is the use of media data for tracking. For the detection of return to competition, minutes played, number of matches and level of play, official websites of the respective associations and leagues were used. Not documented matches, e.g. friendly matches, are therefore not included in the data collection. Nevertheless, the use of media data has already been validated for football [12, 13, 27]. In addition to the ACL rupture, concomitant injuries were not registered and analysed separately. Another limitation is caused by the design of the study. No player surveys or tests were carried out on players, so that no statement can be made about the reasons for the reduced playing time or decline in the playing class.

The results of RTC in different levels of performance are relevant for orthopaedic surgeons, physiotherapists, team physicians, trainer and the player themselves for having a clear summary on the long rehabilitation time after ACL injury in their playing level. In the case of amateur players, this can provide a rationale for an extended return to competition and a more extended rehabilitation period. In particular, the reduction in performance shows the severity of the injury and the long recovery, even for non-medical professionals.

## Conclusion

Decreasing level of play results in a prolonged absence from match activity in football after anterior cruciate ligament ruptures. The trend continues in the rate of career ends and relegation of playing level. In semi-professional and amateur football players in Germany, only minority can hold their level of play compared to the pre-injury level. Simultaneously the match performance, measured by played minutes and matches, was only in professional athletes 2 years after injury at the pre-rupture level, while semi-professionals and amateurs could not reach the same level within 3 years.
